# Importance of Mesoporous Silica Particle Size in the Stabilization of Amorphous Pharmaceuticals—The Case of Simvastatin

**DOI:** 10.3390/pharmaceutics12040384

**Published:** 2020-04-22

**Authors:** Justyna Knapik-Kowalczuk, Daniel Kramarczyk, Krzysztof Chmiel, Jana Romanova, Kohsaku Kawakami, Marian Paluch

**Affiliations:** 1 Faculty of Science and Technology, Institute of Physics, University of Silesia, SMCEBI, 75 Pułku Piechoty 1a, 41-500 Chorzów, Poland; 2 International Center for Materials Nanoarchitectonics, National Institute for Materials Science, Tsukuba, Ibaraki 305-0044, Japan; 3 Department of Physical Chemistry, Faculty of Chemical Technology, University of Pardubice, Studentská 573, 532 10 Pardubice, Czech Republic

**Keywords:** simvastatin, amorphous pharmaceuticals, mesoporous silica, stabilization, recrystallization

## Abstract

In this paper, the role of mesoporous silica (MS) particle size in the stabilization of amorphous simvastatin (SVT) is revealed. For inhibiting recrystallization of the supercooled drug, the two MS materials (Syloid^®^ XDP 3050 and Syloid^®^ 244 FP) were employed. The crystallization tendency of SVT alone and in mixture with the MS materials was investigated by Differential Scanning Calorimetry (DSC) and Broadband Dielectric Spectroscopy (BDS). Neither confinement of the SVT molecules inside the MS pores nor molecular interactions between functional groups of the SVT molecules and the surface of the stabilizing excipient could explain the observed stabilization effect. The stabilization effect might be correlated with diffusion length of the SVT molecules in the MS materials that depended on the particle size. Moreover, MS materials possessing different particle sizes could offer free spaces with different sizes, which might influence crystal growth of SVT. All of these factors must be considered when mesoporous materials are used for stabilizing pharmaceutical glasses.

## 1. Introduction

The poor aqueous solubility of active pharmaceutical ingredients (APIs) is one of the most challenging issues of modern pharmacy [1,2,3]. Currently, over 40% of marketed immediate-release oral dosage forms contain poorly soluble drugs [4,5]. One of the most efficient methods that can improve solubility of poorly soluble drugs is amorphization [6,7,8]. It has been many times reported that the transformation into amorphous form significantly increased the solubility of drug molecules in comparison with their crystalline counterparts [8,9]. These benefits, however, come at a risk. The high internal energy of amorphous solids, which, on the one hand, is the reason for their high solubility, on the other hand, makes amorphous materials thermodynamically unstable [10,11,12,13,14]. Thus, currently, much effort is being made to (i) investigate physical stability of amorphous form of pharmaceuticals [15,16,17], (ii) find effective methods leading to their stabilization [18,19,20], and (iii) discover the molecular mechanisms responsible for the observed recrystallization inhibition [16,21,22,23,24,25].

As has been recently proven, one of the very effective inhibitors for recrystallization of the amorphous APIs during the time of their storage, transportation, or manufacturing are mesoporous silica (MS) materials [17,26,27,28]. It is worth highlighting that MS materials seem to be ideal excipients for drug formulation. This is because they might very effectively stabilize amorphous APIs and they can also very effectively enhance their bioavailability [29]. A great example of a drug in which bioavailability has been effectively enhanced after preparation MS based formulation is fenofibrate [30]. In choosing an appropriate MS for drug formulation, it is important to check its degradability. This is mainly because the approved pharmaceutical products must not accumulate in the human body since it can lead to unpredictable side-effects [31]. It has been proven that various biodegradable MSs are characterized by different speeds of biodegradability. This is a huge advantage of MS, since it results in the possibility of tuning the material to the selected drug according to the targeted applications [32].

Usually, the improvement of an amorphous drug’s physical stability by MS is explained by one of two mechanisms: (i) confinement of the API molecules inside the MS pores or (ii) molecular interactions between functional groups of the API molecules and the surface of the stabilizing excipient [28,33,34]. It is worth noting that, in the case of the former mechanism, it is possible to reach even an eternal stabilization effect [35]. Such a situation might occur only when the pore diameter of the employed MS is smaller than the critical crystal nuclei of the API, as well as if all API molecules are incorporated inside the pores. When the drug molecules are present outside the MS pores, the stabilization is usually explained by the second mechanism [36,37]. MS materials can inhibit the recrystallization of disordered APIs through interactions between the functional groups of the drug molecules and those on the MS surface; this is mainly due to their large specific surface area, which is often larger than 300 m^2^/g [38]. It has to be pointed out that this stabilization mechanism has one limitation—it works only when amount of the MS is enough to host a few layers of API molecules. In other words, if the number of drug molecules exceeds the amount of drug that can be “immobilized” on the MS surface, this mechanism cannot work for the inhibition of drug recrystallization. To accurately determine the loading capacity of a drug on MS surface, one can employ the method found by Hempel et al. (2019), which is an extension of the principle proposed by Mellaertes et al. (2017) [39,40]. This method is based on quantification of the API fraction that has not been immobilized by the MS surface through the detection of a glass transition temperature by Differential Scanning Calorimetry (DSC).

In both stabilization mechanisms mentioned above, pores size, pore-volume, and surface area of MS play crucial roles. Consequently, one can find plenty of information on how these parameters affect the physical stability of amorphous APIs [41,42,43]. Little is known, however, about how the physical stability of amorphous APIs is influenced by the particle size of MS. Thus, the main aim of this article was to investigate the effect of the particle size of MS on the physical stability of amorphous API. As a model drug, we chose simvastatin (SVT)—a commonly prescribed lipid-lowering medication. This pharmaceutical is characterized by excellent permeability but exhibits poor, solubility-limited, bioavailability (5%) [44]. Therefore, there is a need to improve the solubility of this compound. Two MS materials with a brand name of Syloid^®^ 244 FP (SYL244) and Syloid^®^ 3050 XDP (SYL3050) have been employed for stabilizing amorphous state of SVT. These materials are characterized by nearly the same pore size, pore-volume, and surface area (see Table 1) [45,46] but differ in particle size. SYL3050 has an order of magnitude bigger particles than SYL244. To examine the tendency toward recrystallization of SVT mixed with MS materials, time-dependent isothermal crystallization experiments were performed utilizing two different experimental techniques: Differential Scanning Calorimetry (DSC) and Broadband Dielectric Spectroscopy (BDS). The principles of BDS are comprehensively reviewed in the book edited by Kremer and Schönhals (2003) [47]. The utilization of this experimental technique to study molecular mobility and crystallization phenomena in pharmaceutical systems are explained in detail in Grzybowska et al. [48] as well as in the books edited by Rams-Baron and Descamps [49,50]. The principles of DSC have been discussed in detail in Watson et al. (1964) [51] and Höhne et al. (2003) [52]. The use of DSC in the investigation of the isothermal cold crystallization of amorphous APIs has been briefly presented in Szklarz et al. and Kolodziejczyk et al. [13,53]. Since all performed experiments showed that particle size had a significant impact on the physical stability of supercooled SVT, we tried to find the molecular mechanism responsible for the observed recrystallization inhibition.

## 2. Materials and Methods 

### 2.1. Materials

Simvastatin (SVT) with purity higher than 99.3% and molecular mass M_w_ = 418.6 g/mol was purchased from Polpharma (Starogard Gdański, Poland). This pharmaceutical is described chemically as Butanoic acid, 2,2-dimethyl-(1S,3R,7S,8S,8aR)-1,2,3,7,8,8a-hexahydro-3,7-dimethyl-8- [2-[(2R,4R)-tetrahydro-4-hydroxy-6-oxo-2H-pyran-2-yl]ethyl]-1-naphthalenyl ester. Syloid^®^ XDP 3050 (SYL3050) and Syloid^®^ 244 FP (SYL244), with the detailed specification presented in Table 1, were received as a gift from Grace GmbH & CO. KG (Worms, Germany). All chemicals were used as received.

### 2.2. Sample Preparation

In order to obtain the binary mixtures containing simvastatin and 9, 18, 27, 36, 45, and 50 wt. % of SYL3050 or SYL244 the right amount of ingredients was weighed and mixed in mortars for about 10 min. Prior to each experiment, the simvastatin in the physical mixture was melted at 423 K and quenched. For DSC experiments the sample was vitrified in situ the machine (with the flow of N2 = 60 mL/min and cooling rate = 20 K/min), while for dielectric and microscopic experiments, the melting procedure takes place at the hot plate in air conditions. Melted material that was placed between the stainless-steel plates of the capacitor (for BDS) or glassy plates (for the optical microscope) was cooled by a cold cooper plate with a rate of ca. 60 K/min.

### 2.3. Differential Scanning Calorimetry (DSC)

Thermal properties of SVT alone and that with SYL244 or SYL3050 were examined by a Mettler–Toledo DSC 1 STAR^e^ System (Columbus, OH, USA) equipped with an HSS8 ceramic sensor and 120 thermocouples. The instrument was calibrated for temperature and enthalpy using indium and zinc standards. Melting point was determined as the onset temperature, whereas the glass transition temperature as the midpoint of the heat capacity increment. The samples were measured in an aluminum crucible (40 μL). During non-isothermal experiments, heating rate of 10 K/min was employed. Each non-isothermal experiment was repeated three times, while isothermal experiments were repeated twice.

### 2.4. Broadband Dielectric Spectroscopy (BDS)

Molecular dynamics of SVT alone and with SYL244 or SYL3050 was measured with a Novocontrol GMBH Alpha dielectric spectrometer (Montabaur, Germany). Dielectric spectra were registered in a broad frequency range from 10^−1^ Hz to 10^6^ Hz. During the dielectric experiments the sample was heated from 173 K to 298 K with a step of 5 K and from 330 K to 362 K with a step of 2 K. The temperature was controlled by a Quattro temperature controller with temperature stability better than 0.1 K. The systems were measured in a parallel-plate cell made of stainless steel (diameter of 20 mm, and a 0.1 mm gap provided by silica spacer fibers).

### 2.5. Optical Microscope

Optical images of SVT alone and the mixtures with 9 wt. % of SYL3050 or 9 wt. % of SYL244 were captured using an Olympus BX51 polarized microscope (Olympus America Inc., Melville, NY, USA) equipped with an Olympus SC30 camera and a halogen source light. Optical images were collected using an Olympus Soft Imaging Solutions GmbH 5.1 (Münster, Germany) (analysis getIT software) at UMPlanFI 10× objective and at 0.3 NA. All images were handled by Adobe Photoshop 12 software (Adobe Systems, San Jose, CA, USA).

## 3. Results and Discussion

### 3.1. Isothermal Crystallization Studies Performed by DSC

**Figure 1:**
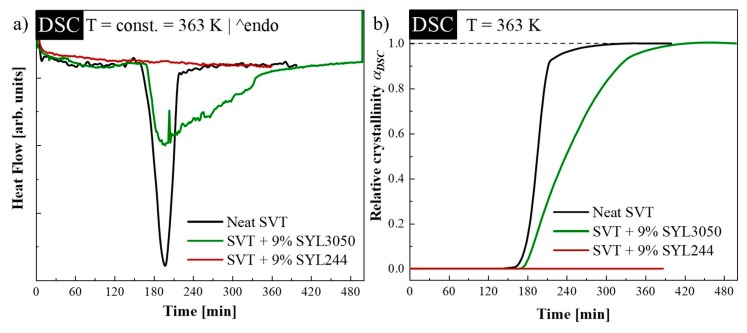
(**a**) Differential Scanning Calorimetry (DSC) traces of neat simvastatin (SVT) (black line), SVT + 9 wt. % of SYL3050 (green line), and SVT + 9 wt. % of SYL244 (red line) recorded during isothermal crystallization at 363 K (**b**) and corresponding relative crystallinity (α_DSC_).

Isothermal crystallization of neat SVT and its mixture with 9 wt. % of SYL244 or SYL3050 was investigated using DSC at 363 K, which is higher than the glass transition temperature by 58 K. Figure 1a shows the representative results obtained during the time-dependent isothermal measurements. The DSC curves of neat SVT and system containing SVT and SYL3050 reveal the exothermic peak of isothermal crystallization. The temperatures for crystallization onset of neat SVT and that for the mixture with SYL3050 were nearly the same (there is ~6 min shift after MS inclusion). However, big difference was observed for the time required for complete crystallization. In the case of neat SVT, the recrystallization ended after 5 h, while the presence of SYL3050 extended this process to 8 h. Interestingly, the presence of the same amount of SYL244, which has a smaller particle size, inhibited crystallization. This result indicates that the particle size of MS might have a significant impact on the physical stability of the amorphous SVT.

Based on data obtained from DSC, one can estimate the relative degree of the sample crystallization (α_DSC_) by utilizing the following formula:

(1) $$\alpha_{DSC}=\frac{\int_{t_{0}}^{t}\frac{dH}{dt}dt}{\int_{t_{0}}^{t_{\infty }}\frac{dH}{dt}dt}$$

where d*H*/d*t* is the rate of heat evolution. *t_0_* and *t_∞_* represent the time at which crystallization begins and ends, respectively. The time evolutions of α_DSC_, as determined from DSC experiments, are presented in Figure 1b. The kinetic curves were normalized by the maximal value of the α_DSC_, which was registered when crystallization has ended. After the isothermal step of DSC experiments, the samples were cooled down and reheated to confirm the degree of crystallinity.

**Figure 2:**
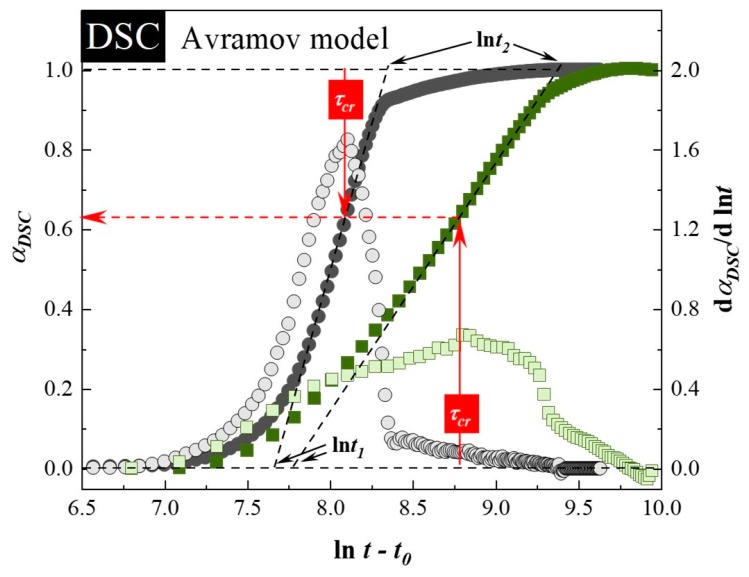
The Avrami–Avramov plot presenting a time evolution of relative crystallinity (α_DSC_) (full symbols) and its first derivative toward the natural logarithm of the time (shadowed symbols) of neat SVT (grey circles) and SVT + 9 wt. % of SYL3050 (green squares).

To properly describe the crystallization kinetics of the investigated samples under isothermal conditions, the Avramov model was employed [54]. In this approach, the dependence of α_DSC_, together with its first derivative, is plotted versus *ln* t on the same axis. In coordinates α_DSC_ against *ln* t, the inflection points in all cases appeared at α < 0.63, and induction times have been determined as 8800 ± 100 s and 9150 ± 50 s for neat SVT and SVT + 9 wt. % of SYL3050, respectively. Finally, i.e., utilizing the value of *t_0_*, the correct Avrami–Avramov plots have been constructed (see Figure 2). From this plot, one can obtain the value of the characteristic time of the crystallization process (*τ_cr_*) as the time corresponds to d(α_DSC_)′/[d(*ln*(t − t_0_))] peak maximum. The determined *τ_cr_* for neat SVT and SVT + 9 wt. % of SYL3050 are equal to 55 ± 1 min and 107 ± 3 min, respectively. The change in *τ_cr_* toward the larger value after addition of SYL3050 indicated improvement in physical stability of SVT in the presence of the MS. Of course, a much better stabilization effect has been reached after employment of the MS characterized by an order of magnitude smaller particle size than in case of SYL3050, what is reflected as lack of SVT re-crystallization.

Use of the Avramov model allows us to calculate another parameter, *n*, which is directly related to the nucleation dimensionality. Two methods are available to determine *n*. The first is based on employment the following equation:

(2) $$n=\frac{{\left(\alpha \left(t\right)\right)}_{max}^{\prime }}{0.368}$$

where (α(*t*))′_max_ is a maximum value of the first derivative of the normalized degree of crystallization. The second approach of evaluation the Avramov parameter related to the nucleation dimensionality is based on drawing a tangent to the experimentally determined sigmoidal curve α_DSC_(*ln*(t − t_0_)) at *t* − *t_0_* = *τ_cr_* (see dashed lines in Figure 2). By determining the values of *ln* t_1_ and *ln* t_2_, which corresponds to the points of intersection of the tangent line with the horizontal straight lines, constructed at the limit values of α_DSC_, i.e., at 0 and 1, it is possible to establish the *n* parameter from the following formula:

(3) $$n=\frac{e}{\ln t_{2}-\ln t_{1}}$$

The values of *t_0_*, *ln* t_1_, *ln* t_2_, (α(*t*))′_max_, *τ_cr_* as well as *n* calculated using both equations are collected in Table 2. As can be seen, regardless of the employed method for determination of *n* value, the dimensionality of crystallization of SVT was reduced when SYL3050 was added to the drug.

### 3.2. Isothermal Crystallization Studies Performed by BDS

**Figure 3:**
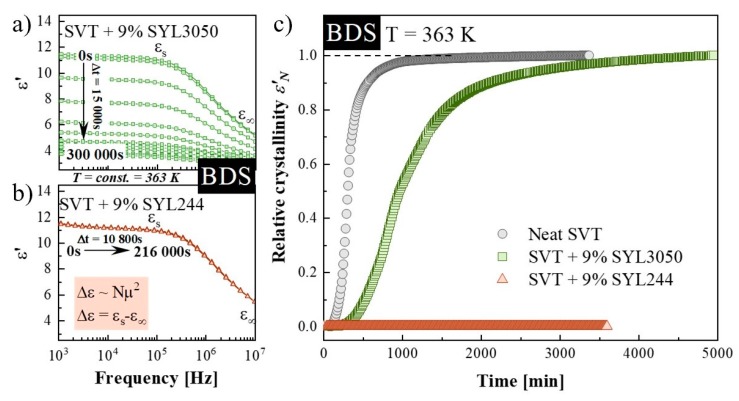
(**a**) Dielectric spectra of the real parts of the complex dielectric permittivity during an isothermal crystallization of SVT + 9 wt. % of SYL3050 performed at 363 K, (**b**) dielectric spectra of the real parts of the complex dielectric permittivity collected during the time-dependent isothermal experiment of SVT + 9 wt. % of SYL244 performed at 363 K, (**c**) normalized dielectric constants ε′_N_ as a function of time from crystallization processes occurring at 363 K.

The second method employed to study the isothermal crystallization of neat SVT and its mixtures with MSs having two types of particle size was BDS. During the time-dependent dielectric experiments, the spectra of the complex dielectric permittivity ε*(ω) = ε′(ω) − iε″(ω) were investigated at specified time intervals of 300 s. By using dielectric spectroscopy, the crystallization process can be followed directly in both the real (ε′) and imaginary (ε″) parts of the complex dielectric permittivity, reflected by a decrease of the static permittivity (ε_s_) and reduction of the loss peak intensity with time, respectively [49]. For our purpose, the real part of complex dielectric permittivity was selected for further analysis. The representative frequency dependences of ε′ measured during the time-dependent dielectric experiments performed at T = 363 K for SVT + 9 wt. % of SYL3050 as well as SVT + 9 wt. % of SYL244 are presented in Figure 3a,b. The neat SVT and that in the mixture with SYL3050 recrystallized as evidenced by the registered decrease in the static permittivity (ε_s_). Lack of drop in the ε_s_ observed during identical measurements performed on the SVT + 9 wt. % of SYL244 system (Figure 3b) indicated that the MS with smaller particle size was a better stabilizer for the amorphous SVT. This investigation agrees with the finding made during the DSC study where the particle size of MS was the important parameter for stabilizing amorphous SVT. After the isothermal dielectric experiments, the neat SVT and its mixture with SYL3050 were subjected to the DSC measurement to confirm that crystallinity of both samples reached 100%.

Usually, the progress of crystallization is analyzed in terms of the normalized real permittivity (*ε*′_*N*_) defined as follows [55,56,57]:

(4) $$\varepsilon_{N}^{\prime }\left(t\right)=\frac{\varepsilon^{\prime }\left(0\right)-\varepsilon^{\prime }\left(t\right)}{\varepsilon^{\prime }\left(0\right)-\varepsilon^{\prime }\left(\infty \right)}$$

where *ε*′(0) is the initial static dielectric permittivity, *ε*′(∞) is the long-time limiting value, and *ε*′(*t*) is the value at time *t*. The data normalized in this way and plotted versus time are shown in Figure 3c.

**Figure 4:**
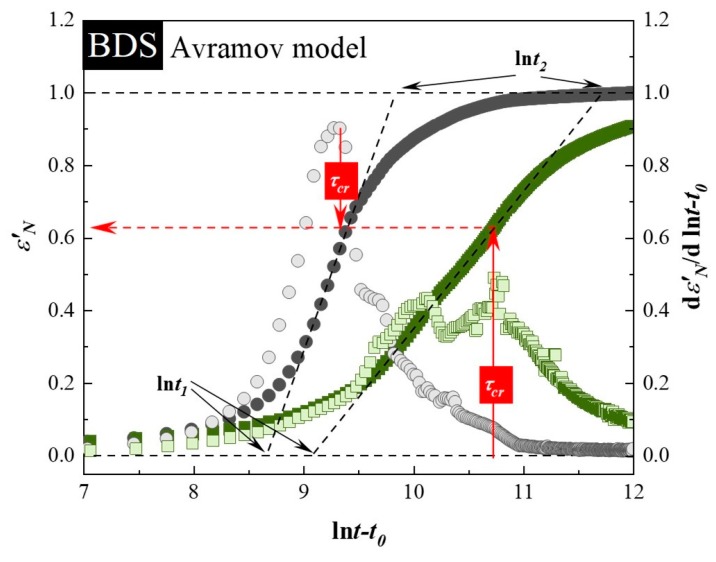
The Avrami–Avramov plot presenting a time evolution of normalized real permittivity (*ε*′_*N*_) (full symbols) and its first derivative toward the natural logarithm of the time (shadowed symbols) of neat SVT (grey circles) and SVT + 9 wt. % of SYL3050 (green squares).

Crystallization of SVT in the mixture with 9 wt. % of SYL3050 is delayed in comparison to that of the neat SVT. The entire crystallization process of the neat SVT and that in the mixture with SYL3050 required ca. one day and three days, respectively, at 363 K. Analysis based on the Avramov model revealed that the inflection points have appeared at *α* < 0.63, whereas the induction times have been determined as 13,600 ± 400 s and 29,750 ± 250 s for neat SVT and SVT + 9 wt. % of SYL3050, respectively. By utilizing the estimated values of *t_0_*, the Avrami–Avramov plot for each sample was constructed (Figure 4). Determined from the *d(α_DSC_)′/[d(ln t − t_0_)]* peak maximum, the characteristic time of the crystallization process (*τ_cr_*) for neat SVT and that in the mixture with 9 wt. % of SYL3050 are equal to 201 ± 12 min and 737 ± 28 min, respectively.

The *n* values together with other parameters were determined in the same manner as described in the previous section are summarized in Table 3. The *n* value for the SVT in the mixture with SYL3050 is smaller than that for the neat SVT, which agreed with the results obtained from the DSC study.

**Figure 5:**
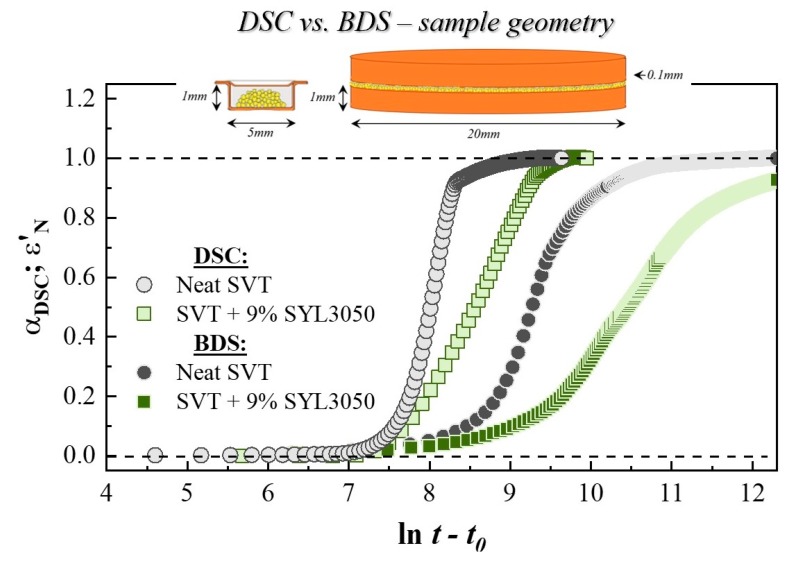
Comparison of the time evolutions of normalized real permittivity (*ε*′_*N*_) and relative crystallinity (α_DSC_) as well as its first derivatives toward the natural logarithm of the time of neat SVT (grey circles) and SVT + 9 wt. % of SYL3050 (green squares).

It is worth highlighting that the crystallization kinetics of SVT was characterized by totally different parameter values for two different experimental techniques [58]. The recrystallization during the BDS measurement was much slower than that in the DSC study. For example, *t_0_*_BDS_ for the SVT in the mixture was 3.25 times longer than *t_0_*_DSC_. By employing the dielectric spectroscopy, one can also observe an increase in the value of *τ_cr_* as well as decrease in the *n* parameter in comparison to the values determined from the DSC study. The described differences between crystallization kinetics of the same systems measured by different experimental techniques are natural and result from differences existing between the employed techniques. For example, samples used for the both techniques had totally different geometry (see inserts in Figure 5). The sample thickness for the BDS study was 0.1 mm, which was much thinner than that in the DSC measurement. The difference in the sample thickness results in different heat flow, which may influence the crystallization kinetics [58]. During the dielectric studies, samples were placed between stainless-steel electrodes, which inhibited their contact with air. A decrease in the specific surface area delays crystallization because nucleation is frequently initiated from the surface [59,60]. Also, in the case of DSC measurements, samples were heated and quenched at a rate of 20 K/min under a nitrogen atmosphere prior to the crystallization experiment, whereas those for BDS studies were melted in the air on a hot plate and quenched at a rate faster by four-times than that for the DSC study. Both the atmosphere and the cooling rate [61] influence the amorphous property. Dimension of crystal growth is also influenced by the sample geometry [59]. Thus, the smaller *n* values in the BDS study compared to those from the DSC study is natural observation. Nevertheless, it should be emphasized that, despite quantitative differences in the crystallization kinetics obtained by the two different experimental techniques, one can find qualitative similarities on effect of the presence of the MS material, that is, its stabilization effect against crystallization of SVT. Moreover, a more striking stabilization effect was observed for SYL244 relative to SLY3050 despite their almost equal surface area (~300 m^2^/g), pore size (~20 nm), and pore volume (~1.65 mL/g). Therefore, it seems essential to find the reason for the observed differences in the stabilization of supercooled SVT. To achieve this goal, we were looking for the differences in the thermal properties and molecular dynamics of these compositions.

### 3.3. Loading Capacity of MSs for SVT

**Figure 6:**
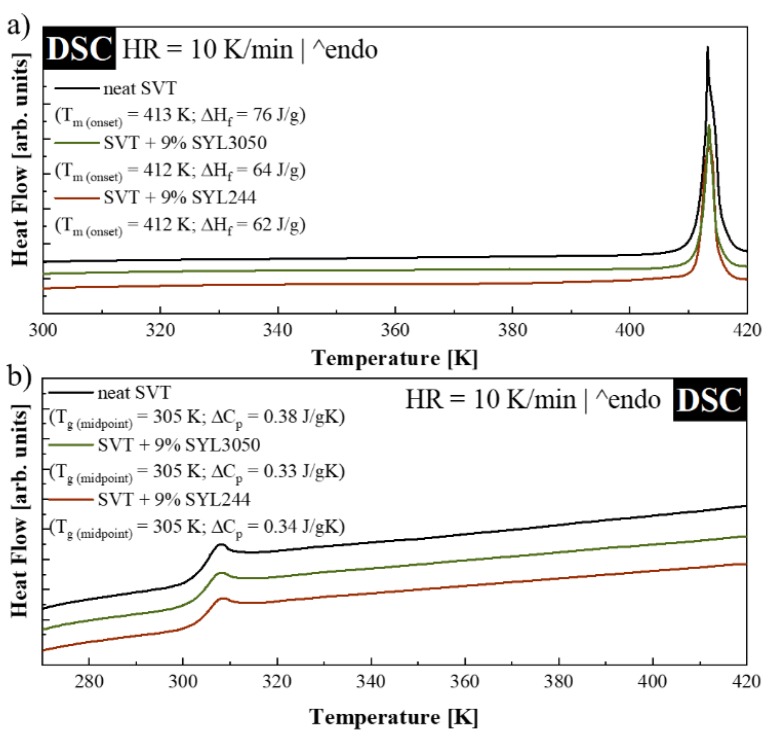
DSC thermograms of (**a**) crystalline and (**b**) amorphous SVT (grey lines), SVT + 9 wt. % of SYL3050 (green lines), and SVT + 9 wt. % of SYL244 (red lines).

To investigate how the MS materials, possessing different particle sizes, influence the thermal properties of SVT, both physical mixtures (i.e., samples containing crystalline API) and quenched samples (i.e., samples containing amorphous API) have been investigated non-isothermally through DSC. Figure 6a presents the DSC thermograms obtained during the sample heating with a rate equal to 10 K/min. As can be seen, neither the melting temperature of SVT nor its glass transition has been significantly modified after the inclusion of MS materials. The melting temperature of neat SVT, determined as the onset of the registered melting endotherm, is equal to 413 K. Mixtures containing SVT and 9 wt. % of SYL244 or SYL3050 are characterized by *T_m_* equal to 412 K. After the quenching of all samples, the reheating DSC curves were acquired. As can be seen in Figure 6b, the thermogram of each sample reveal one step-like thermal event corresponding to glass transition of SVT. The *T_g_* midpoints of neat SVT, SVT + 9 wt. % of SYL244, and SVT + 9 wt. % of SYL3050 have the same value that is equal to 305 K, when heated at a rate 10 K/min.

**Figure 7:**
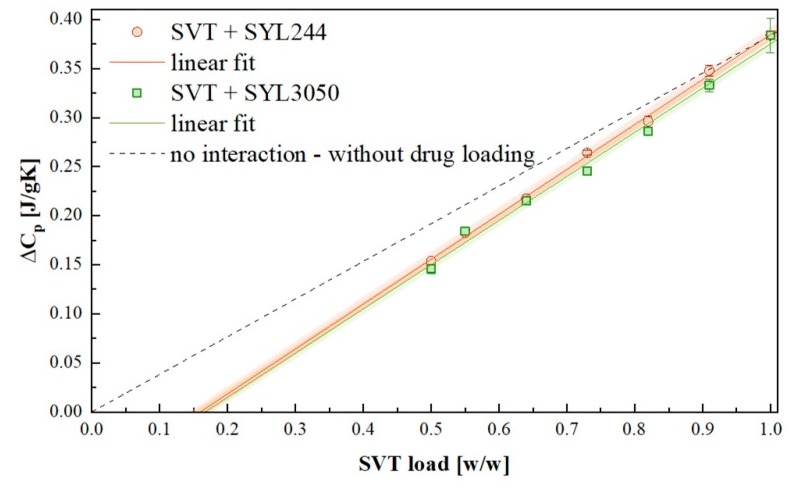
Linear extrapolation of the obtained Δ*C_p_* as a function of drug load.

After the inclusion of MS material to SVT, the value of its Δ*C_p_* decreased. In the ideal case in which SVT molecules would not interact with the surface of MS, the value of Δ*C_p_* of the mixture should have linear relationship with the amount of SVT. When, however, some interactions between the drug and surface of MS exist, the decrease of Δ*C_p_* is larger than expected. Recently, Hempel et al. showed that by measuring the Δ*C_p_* value of various concentrations of a system containing drug and MS material, it is possible to estimate the monomolecular loading capacity of the drug on the surface of MS [41]. A series of samples possessing various concentrations of MS and SVT have been prepared and investigated in the same manner. The concentration dependences of Δ*C_p_* of SVT are presented in Figure 7.

By extrapolating a straight line describing the concentration dependence of Δ*C_p_* of both SVT + SYL244 and SVT + SYL3050 systems to zero, the monomolecular loading capacity values were determined. The amount of MS materials required to stabilize all SVT molecules on their surface was equal to 84.3 wt. % and 83.4 wt. % for SYL244 and SYL3050, respectively. Lack of significant discrepancies between these values proved that the employed MS materials interacted similarly with SVT. Therefore, considering these results, it is difficult to explain the dramatic difference in the stabilization effect of MS for the amorphous SVT by their loading capacities.

### 3.4. Effect of MS Materials on the Molecular Mobility of Supercooled SVT

**Figure 8:**
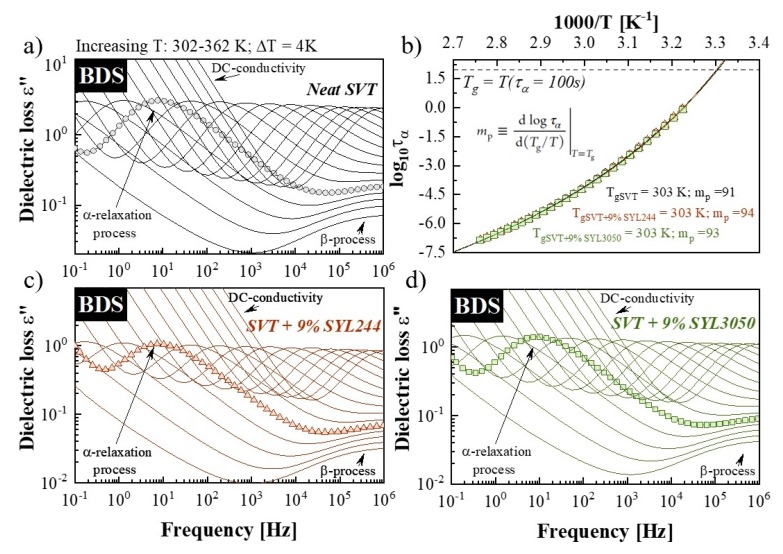
Dielectric loss spectra of (**a**) neat SVT, (**c**) SVT + 9 wt. % of SYL244, and (**d**) SVT + 9 wt. % of SYL3050 collected above their respective *T_g_*s upon heating. In panel (**b**), activation plots are constructed for the tested compounds with gray circles, red triangles, and green squares referring to temperature dependences of *α*-relaxation times for neat SVT, SVT + 9 wt. % of SYL3050, and SVT + 9 wt. % of SYL244, respectively. The solid lines are the fitting results by the Vogel−Fulcher−Tammann (VFT) equation.

Since no significant differences in loading capacities have been found between two MS materials, the following questions arise: Is the observed difference in SVT stabilization by MS materials possessing different particle size associated with some modifications in dynamics of the drug molecules? Is there any difference in the τ_α_(T) of SVT when different MSs are employed? Or does the inhibition of the secondary relaxation processes play a crucial role? To answer these questions, molecular dynamics of the neat SVT and the systems containing SVT + 9 wt. % of SYL3050 and SVT + 9 wt. % of SYL244 were evaluated by means of BDS. Representative dielectric loss spectra, which were measured above the samples glass transition temperatures, are presented in Figure 8a,c,d. As can be seen at this temperature region, the spectra of all investigated samples exhibit two features—the dc-conductivity related to translational motions of ions and the structural (α) relaxation process associated with the cooperative rearrangement of the entire molecules. The α-relaxation mode always shifts toward higher frequencies with increasing temperature, indicating an increase in global mobility of the systems.

From the analysis of dielectric loss spectra registered at supercooled liquid state, the temperature dependences of structural relaxation time (*τ_α_*(*T*)) of all investigated samples were determined (see Figure 8b). To obtain the value of *τ_α_* at various temperature conditions, we fitted the experimental data by the Havriliak–Negami (HN) function. The empirical HN approach with the dc-conductivity term is given by the following formula [62]:

(5) $$\varepsilon^{*}\left(\omega \right)=\varepsilon_{\infty }+\frac{\Delta \varepsilon }{{\left[1+{\left(i\omega \tau_{HN}\right)}^{a}\right]}^{b}}+\frac{\sigma_{dc}}{\varepsilon_{0}i\omega }$$

where *ε_∞_* is the high-frequency limit permittivity, *ε_0_* denotes the permittivity of vacuum, Δ*ε* is dielectric strength, *ω* is equal to 2πf, *τ_HN_* is the HN relaxation time, and *a* and *b* represent symmetric and asymmetric broadening of the relaxation peak. Employing the fit parameters determined above, we finally calculated the values of *τ_α_* as

(6) $$\tau_{\alpha }=\tau_{max}=\tau_{HN}{\left[\sin(\frac{\pi ab}{2+2b})\right]}^{-\frac{1}{a}}{\left[\sin(\frac{\pi ab}{2+2b})\right]}^{\frac{1}{a}}$$

In the supercooled liquid region, the temperature evolution of *τ_α_* usually shows non-Arrhenius behavior. Thus, to properly described *τ_α_*(*T*) dependences of neat SVT and its mixture with 9 wt. % of SYL244 or SYL3050 we employed the Vogel−Fulcher−Tammann (VFT) equation that is expressed as follows [63,64,65]:

(7) $$\tau_{\alpha }=\tau_{\infty }\exp\left(\frac{DT_{0}}{T-T_{0}}\right)$$

where *τ_∞_*, *T_0_*, and *B* are fitting parameters. Parameter *τ_∞_* is a pre-exponential factor denoting the upper limit of temperature for *τ_α_*, which is correlated to vibrational frequency (∼10^−11^ to 10^−14^ s). *T_0_* is the Vogel temperature, which correspond to the state with infinite relaxation time, and *D* denotes deviation from the Arrhenius model. Extrapolating the VFT fits to temperature at which *τ_α_* = 100 s, the *T_g_* values of all the samples have been estimated to be 303 K. The glass transition temperatures determined by this method are in good agreement with that obtained from calorimetric studies (*T_gDSC_*_HR =10 K/min_ = 305 K—see Figure 6). From the VFT fits, we also calculated the value of fragility parameter, *m_p_*, for all investigated samples. This parameter is a measure of deviation the *τ_α_*(*T*) dependence from the Arrhenius behavior, and is defined as [66]

(8) $${m_{p}=\frac{d\log\tau_{\alpha }}{d\left(\frac{T_{g}}{T}\right)}|}_{T=T_{g}}$$

The typical values of the fragility parameter are between 50 and 100 [22,67,68]. The higher the fragility value, the more fragile the liquids. The *m_p_* parameter is considered to help predict the physical stability of amorphous pharmaceuticals because it has been implied that strong materials are more stable than the fragile ones [61,69]. However, the addition of MS did not have much of an impact on *m_p_* (Table 4); therefore, the difference in the crystallization behavior cannot be explained by the fragility. Thermodynamic parameters for each amorphous material are collected in Table 4. As can be seen, 9 wt. % of the used MS materials do not significantly modify the temperature evolution of τα of SVT, and consequently, no explanation of the observed stabilization has yet been found.

**Figure 9:**
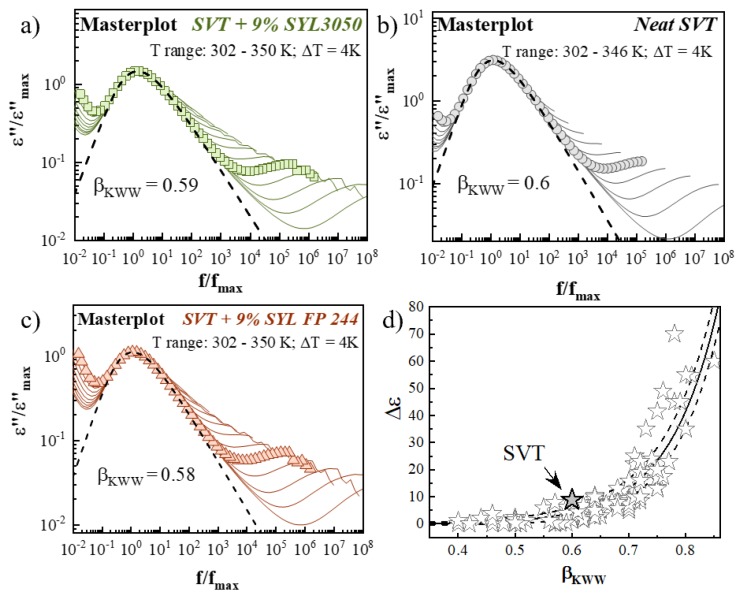
(**a**–**c**) The master plot dielectric loss spectra of SVT + 9 wt. % of SYL3050, neat SVT, and SVT + 9 wt. % of SYL244 formed by horizontal shifting of spectra to overlap the reference one. The dashed lines represent the KWW fit to the *α*-peak at 314 K with *β_KWW_* = 0.6, 0.59, and 0.58 for neat SVT, SVT + 9 wt. % of SYL3050 and SVT + 9 wt. % of SYL244, respectively. (**d**) Dielectric strength Δε(T_g_) as a function of the fractional exponent β_KWW_ in the Kohlrausch-Williams-Watts function, taken from the Reference [76].

To check if the shape of the structural relaxation peak of SVT remains constant in the whole examined temperature range, as well as what impact on it have the employed MS excipients, a so-called master plot has been constructed for each sample (see Figure 9a–c). To obtain the master plot, dielectric spectra taken from 302 K to 350 K was shifted to superimpose on the reference spectrum at 314 K. The master plots show that the shape of the *α*-relaxation of SVT is invariant to the temperature changes, and the parameter *β_KWW_* for all spectra is the same. The value of the *β_KWW_* parameter of SVT, which describes the breadth of its structural relaxation loss peak, was determined by fitting the *α*-peak at a temperature *T* = 314 K through the one-side Fourier transform of the Kohlrausch−Williams−Watts (KWW) function [70]. This procedure gives a value of *β_KWW_* equal to 0.60, 0.59 and 0.58 for neat SVT, SVT + 9 wt. % of SYL3050, and SVT + 9 wt. % of SYL244, respectively. It should be mentioned that the value of *β_KWW_* may vary within the 0–1 range. This parameter approaches 1 if the *α*-relaxation peak is narrow and symmetric and corresponds to the Debye case; however, when its value is approaching 0, the structural relaxation process is broad and asymmetric [47]. The *β_KWW_* might be correlated with crystallization tendency of amorphous materials [71]. It has been suggested that the physical stability of amorphous materials stored at similar relaxation times (*τ_α_*) should decrease as *β_KWW_* increases. Based on this assumption, the physical stability of SVT should not be improved after the addition of MS, although the difference in *β_KWW_* is only marginal. 

According to the recent study by Paluch et al., anticorrelation between the width of the *α*-loss peak and polarity of the molecule, van der Waals glass formers with a broad *α*-loss peak (i.e., a small value of *β_KWW_*) should exhibit a low value of the dielectric strength (Δ*ε_α_*) [72]. SVT with *β_KWW_* = 0.6 and Δ*ε_α_* = 8.9 follows well this anticorrelation similarly to chloramphenicol (*β_KWW_* = 0.8, Δ*ε_α_* = 55) [73], MD20 (*β_KWW_* = 0.76, Δ*ε_α_* = 39) [74], azithromycin (*β_KWW_* = 0.52, Δ*ε_α_* = 1.2), or roxithromycin (*β_KWW_* = 0.62, Δ*ε_α_* = 1.6) [75] (see panel Figure 9d).

### 3.5. Effect of MS Materials on the Molecular Mobility of Glassy SVT

**Figure 10:**
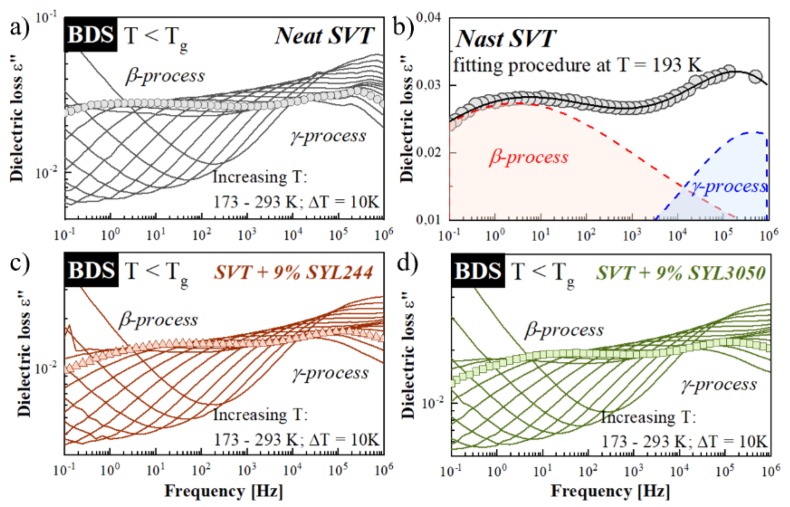
The dielectric loss spectra of (**a**) neat SVT, (**c**) SVT + 9 wt. % of SYL244, and (**d**) SVT + 9 wt. % of SYL3050 registered at temperatures below *T_g_*. (**b**) Selected spectrum of neat SVT with two well-visible secondary relaxation processes *β* and *γ.*

In the glassy state, where the structural—*α*—relaxation becomes too slow to be experimentally observed, it is possible to monitor faster secondary relaxation processes associated with the local (inter- or intramolecular) motions [76]. It has been many times reported that this kind of motion might be responsible for the crystallization of amorphous materials. The best examples of APIs in which secondary relaxations play a crucial role in physical stability are celecoxib and sildenafil [77,78]. To investigate how the MSs materials affect the secondary relaxation of SVT, the dielectric spectra at temperatures 173–293 K have been measured utilizing BDS. Representative spectra for neat SVT, SVT + 9 wt. % of SYL244, and SVT + 9 wt. % of SYL3050 are shown in Figure 10a,c,d.

**Figure 11:**
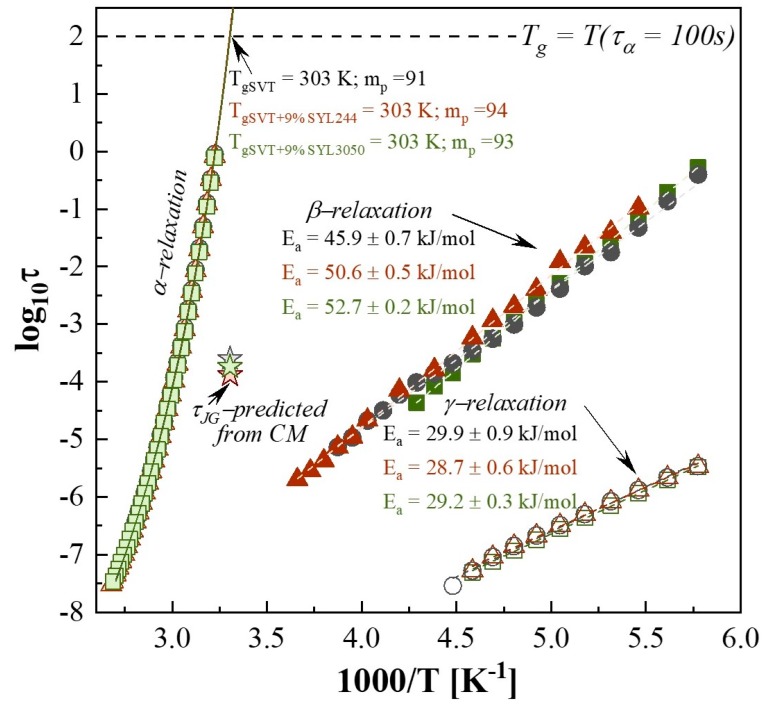
The relaxation map of neat SVT (gray points), SVT + 9 wt. % of SYL244 (red points), and SVT + 9 wt. % of SYL3050 (green points). The Vogel–Tammann–Fulcher (VTF) equation was applied to describe structural relaxation times, while the temperature dependences of secondary relaxation times were fitted to the Arrhenius equation.

Two secondary relaxations (*β* and *γ*) were observed for both the neat SVT and that in mixtures with SYL244 or SYL3050. Both modes move toward higher frequencies with increasing temperature, indicating an increase in molecular mobility. To determine the values of *τ_β_* and *τ_γ_*, the spectra of each sample have been fitted by two Cole−Cole (CC) functions. The example of the performed fitting procedure is presented in panel b of Figure 10. It is worth recalling that the CC function is a special case of the HN function (Equation (5)) in which the *b* parameter is fixed at 1. As Figure 11 presents, in the glassy state of SVT, both *τ_β_*(*T*) and *τ_γ_*(*T*) exhibit a linear dependence, and consequently can be well described by the Arrhenius equation:

(9) $$\tau_{\beta }\left(T\right)=\tau_{\infty }\exp\left(\frac{E_{a}}{RT}\right)$$

where *R* is the gas constant, *τ_∞_* is the pre-exponential factor, and *E_a_* is an activation energy. The obtained values of *E_a_* are collected in Figure 11. However, this analysis revealed that the stabilization effect (exerted) by MS, especially SYL244, cannot be explained by the secondary relaxation as it does not modify the *γ*-relaxation and the fact that it barely changes the dynamics of *β*-process of SVT.

### 3.6. Mechanism of SVT Stabilization with MS Materials

Crystallization of the SVT should be inhibited if it is strongly adsorbed on the surface of MS. In fact, the adsorbed SVT molecules did not exhibit even the glass transition behavior. However, it was obviously not enough to explain the stabilization mechanism. Extensive BDS and DSC analysis revealed that many parameters to describe macroscopic thermodynamic and dynamic properties of the amorphous SVT remained almost the same after the addition of the MS materials. Moreover, despite significant difference in the stabilization effect between SYL244 and SLY3050, their influences on the amorphous properties of SVT was not obvious.

We have added only 9 wt. % of MS to observe the drastic stabilization effect of the amorphous SVT. To provide sufficient loading capacity for SVT, a much larger amount of MS material, ca. 84 wt. %, is required. Consequently, the only remaining difference to explain observed stabilization effect is the difference in the particle size of the MS.

Note that the stabilization effect was observed at 363 K, which is higher than the glass transition temperature by 60 K. Very high molecular mobility is expected for SVT at the experimental temperature for the crystallization study. A very small amount of stabilizers may influence the entirety of the materials because of the rapid diffusion of the SVT molecules. If the particle size is small, the exchange of the SVT molecules in the pores and those outside the particles should occur easily. If the particle size is large, the exchange may become difficult for the molecules located deep in the particles. This may explain the different stabilization effect of the two MS materials with different particle size.

**Figure 12:**
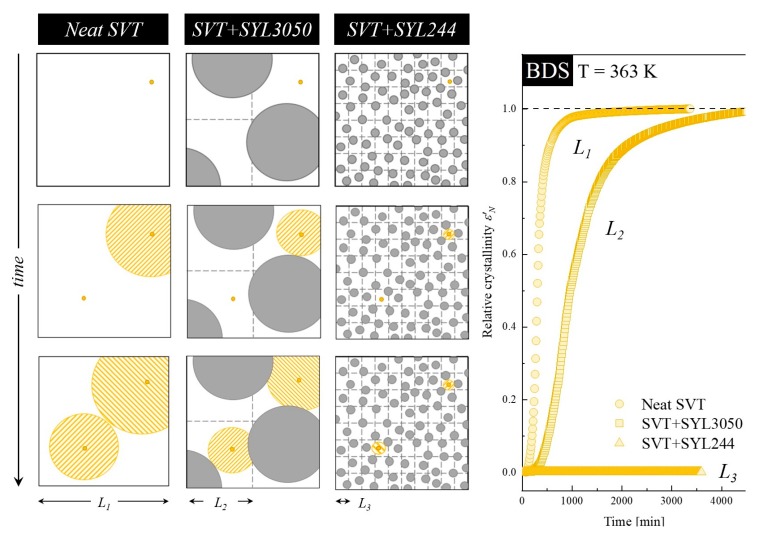
The time evolution of crystallinity of neat SVT (circles), SVT + 9 wt. % of SYL3050 (squares), and SVT + SYL244 (triangles), which were obtained from dielectric studies and described in Section 3.2, together with the schematic explanation of the stabilization mechanism by MS materials. Yellow dot, yellow patterned circles, and gray filled circles represent SVT nuclei, SVT crystals, and MS particles, respectively.

The global crystallization observed through *X*(*t*) (i.e., *α_DSC_*(*t*) or *ε′_N_*(*t*)) consists of nucleation and crystal growth. By a dimensional analysis of three-dimensional nucleation having the nucleation rate *N = L*^−3^*t*^−1^ and the linear crystal growth rate *V* = *Lt*^−1^, one can describe the crystallization process using a characteristic time *t*_0_ = (*NV*^3^)^−1/4^ and a characteristic size *ξ* = (*V*/*N*)^1/4^. As explained by Descamps and Willart [79], the competition between the characteristic, natural length scale (*ξ*), and the real macroscopic size (*L*) of the system induces a change in the kinetic regime as discussed in references [79,80] and visualized in Figure 12.

Particles of SYL244 (MS that is characterized by smaller particle size than SYL3050) may limit the real size of the drug (*L*) more effectively than large particles of SYL3050. On the other hand, particles of SYL3050 can form a restriction in SVT space that is absent when SVT is alone. Such a modification in the sample size can affect both the time scale of the crystallization process and the expression of the kinetic law itself. Consequently, a dramatic slowing down of the SVT kinetic is expected after the reduction of L that is realized by the employment of MS materials.

**Figure 13:**
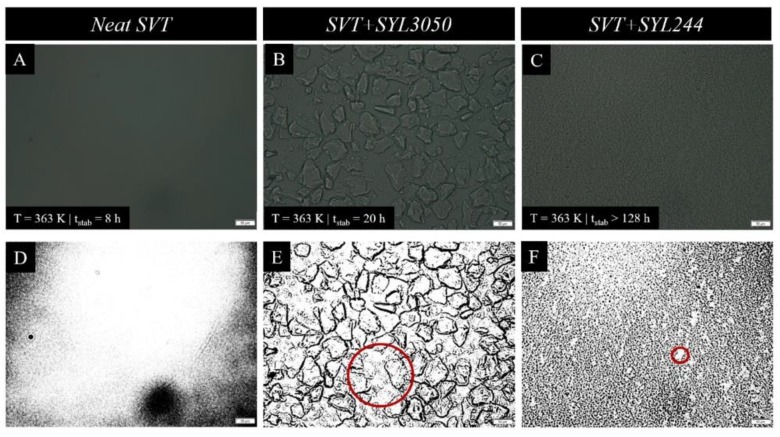
(**A**–**C**) The optical images, which were collected at 5× magnification, of neat SVT, SVT + 9 wt. % of SYL3050, and SVT + SYL244 (the scale bars are equal to 50 µm). (**D**–**F**) The images from panels A–C with artificial contrast (the red circles represent the representative free areas of the SVT alone and in mixture with MS).

To verify the proposed hypothesis explaining the physical improvement of supercooled SVT after the inclusion MS materials, the optical microscopy was employed. The obtained optical images, with a scale bar equal to 50 µm are presented in Figure 13. Panels A–C present the row microscopic data, while panels D–E present improved images with adjustment of the contrast. As can be seen, the smaller the particle size, the more steric hindrance is generated (i.e., less free space for the sample crystal growth exists—compare the areas marked by the red circles in Figure 13E,F). It consequently leads to the reduction of drug connectivity and thereby loss of crystallization propagation pathways and an increase of the physical stability of SVT.

## 4. Conclusions

In this paper, we investigated the effect of two MS materials (SYL244 and SYL3050) on the physical stability of supercooled SVT. These MS materials differ from each other only by the size of particles. SYL3050 possesses particles that are an order of magnitude larger than SYL244. To investigate the kinetics of crystallization of both SVT alone and in mixture with the MS, two experiments—DSC and BDS—were employed. Despite the differences in the obtained crystallization kinetics resulting from the use of different research techniques, one could have observed the same stabilization trends. Neat SVT begins to recrystallize faster, and its crystallization kinetic curve is much steeper than after the inclusion SYL3050. Furthermore, in the case of the MS having a smaller particle size, a lack of sample recrystallization was noted. To find a molecular mechanism responsible for the observed improvement of physical stability of SVT, we performed a series of calorimetric and dielectric studies. The obtained results showed that neither thermal properties nor molecular dynamics are significantly changing after inclusion to SVT the MS material. Consequently, none of the known stabilization mechanisms can explain the observed inhibition of SVT recrystallization. The particle size effect on the stabilization was likely to be explained by difference in exchange process between entrapped and bulk drug molecules. Moreover, reduction in size of the free space for crystal growth might be partially responsible for the different stabilization effect. These additional factors should be considered as well when mesoporous materials are used for stabilizing pharmaceutical glasses in addition to the direct interaction between mesoporous materials and drug molecules.

## Figures and Tables

**Table 1:** Surface chemistry characterization of SYL244 and SYL3050 [47,48].

MS Name:	BATCH/LOS:	Surface Area (m^2^/g)	Average Particle Size (μm)	Pore Diameter (nm)	Pore Volume (mL/g)
SYL244	1000320678	314	2.5–3.7	23	1.6
SYL3050	1000298877	320	59	22.9	1.7

**Table 2:** Comparison of parameters estimated from Avramov model for kinetics of isothermal crystallization obtained from DSC measurements.

Sample:	*t_0_* (s)	*τ_cr_* (min)	*ln* t_1_	*ln* t_2_	*n* (Equation (3))	α(*t*)′ _max_	*n* (Equation (2))
neat SVT	8800 ± 100	55 ± 1	7.65 ± 0.02	8.361 ± 0.001	3.8 ± 0.1	1.57 ± 0.09	4.3 ± 0.2
SVT + SYL3050	9150 ± 50	107 ± 3	7.79 ± 0.04	9.33 ± 0.08	1.8 ± 0.1	0.73 ± 0.06	2.0 ± 0.2

**Table 3:** Comparison of parameters estimated from Avramov model for kinetics of isothermal crystallization obtained from dielectric measurements.

Sample:	*t*_0_ (s)	*τ_cr_* (min)	l*n*1	l*n*2	*n* (Equation (3))	α(*t*)max’	*n* (Equation (2))
neat SVT	13,600 ± 400	201 ± 12	8.71 ± 0.06	9.88 ± 0.02	2.34 ± 0.07	0.91 ± 0.01	2.48 ± 0.04
SVT + SYL3050	29,750 ± 250	737 ± 28	9.02 ± 0.08	11.67 ± 0.08	1.026 ± 0.003	0.488 ± 0.001	1.326 ± 0.001

**Table 4:** Comparison of the obtained based on the dielectric data values of *T_g_*, *m_p_* and fitting parameters from the VFT for neat SVT, SVT + SYL3050, and SVT + SYL244.

Sample:	*T_g_* (K)	log *τ*_∞_	*T*_0_ (K)	BT_0_	*m*_p_
SVT	303	−15.68 ± 0.13	244.01 ± 0.89	2386 ± 51	91
SVT + SYL3050	303	−15.23 ± 0.11	246.23 ± 0.77	2240 ± 42	93
SVT + SYL244	303	−15.18 ± 0.16	247.64 ±1.13	2183 ± 54	94
